# Brassinolide promotes interaction between chloroplasts and mitochondria during the optimization of photosynthesis by the mitochondrial electron transport chain in mesophyll cell protoplasts of *Arabidopsis thaliana*

**DOI:** 10.3389/fpls.2023.1099474

**Published:** 2023-04-11

**Authors:** Kandarpa Mahati, Kollipara Padmasree

**Affiliations:** Department of Biotechnology and Bioinformatics, School of Life Sciences, University of Hyderabad, Gachibowli, Hyderabad, India

**Keywords:** brassinolide, chloroplastic photosynthesis, mitochondrial respiration, cytochrome oxidase and antimycin A, alternative oxidase and salicylhydroxamic acid, malate valve, reactive oxygen species and antioxidant system

## Abstract

The current experimental data unveils the role of brassinolide (BL), a phytohormone of class brassinosteroids (BRs), in augmenting the cross-talk between the mitochondrial electron transport chain (mETC) and chloroplasts to strengthen the efficiency of the Calvin-Benson cycle (CBC) for higher assimilation of carbon dioxide in the mesophyll cell protoplasts (MCP) of *Arabidopsis thaliana*. The outcome of total respiration (TR) and photosynthetic carbon assimilation (PCA) was monitored as O_2_ uptake under dark and NaHCO_3_-dependent O_2_ evolution under light, respectively, after pre-incubation of MCP at a broad spectrum of BL concentration from 0.05 pM to 5 pM at 25 °C and optimum light intensity of 1000 μmol m^-2^ s^-1^. The addition of optimal concentration (0.5 pM) of BL to MCP stimulated the (i) TR, (ii) PCA, and (iii) *para*-benzoquinone-dependent O_2_ evolution (PSII activity). Further, in response to BL, the enzyme activity or transcript levels of redox-regulated CBC enzymes and glucose-6-phosphate raised considerably. Also, the addition of BL to MCP remarkably accelerated the capacity of the cytochrome oxidase (COX) and alternative oxidase (AOX) pathways concurrently with an increase in total cellular pyruvate and reactive oxygen species (ROS) levels. Besides, malate valve components (Malate, *Chl-MDH*, *M-MDH*) increased in response to BL. At the same time, the cellular redox ratios of pyridine nucleotides (NADPH and NADH) were kept low in the presence of BL. However, BL could not keep up the CBC activity of photosynthesis along with its associated light-activated enzymes/transcripts when mETC through COX or AOX pathway is restricted by antimycin A (AA) or salicylhydroxamic acid (SHAM), respectively. In contrast, adding BL to MCP under restricted mETC showed aggravation in total cellular ROS, pyruvate, malate, and redox ratio of pyridine nucleotides with a concomitant increase in transcripts associated with malate valve and antioxidant systems. These results suggest that BL enhances the PCA by coordinating in cross-talk of chloroplasts and mitochondria to regulate the cellular redox ratio or ROS through the involvement of COX and AOX pathways along with the malate valve and antioxidant systems.

## Introduction

In plant mitochondrial electron transport chain (mETC), the movement of electrons bifurcate at ubiquinone to follow either the cyanide-sensitive cytochrome oxidase (COX) pathway or cyanide-resistant alternative oxidase (AOX) pathway respectively to reduce the molecular oxygen (O_2_) into the water (Moore and Siedow, 1991; Vanlerberghe and McIntosh, 1997; Millar et al., 2011). Electron transport through the COX pathway generates proton gradient and ATP synthesis. In contrast, electron transport through the AOX pathway is not coupled to ATP synthesis, and energy is dissipated as heat (Day and Wiskich, 1995; Siedow and Umbach, 2000; Millenaar and Lambers, 2003; Schertl and Braun, 2014).

In a photosynthesizing cell, the COX and AOX pathways of mETC play an essential role in benefiting photosynthetic carbon assimilation (Raghavendra and Padmasree, 2003; Scheibe, 2004; Noguchi and Yoshida, 2008; Vanlerberghe et al., 2020). The involvement of mETC in the optimization of photosynthesis has been demonstrated using the approach of chemical inhibitors or reverse genetics under various environmental stress conditions such as limiting CO_2_ (Padmasree and Raghavendra, 1999a; Padmasree and Raghavendra, 1999b; Padmasree and Raghavendra, 1999c; Padmasree and Raghavendra, 2001a), high light (Saradadevi and Raghavendra, 1992; Dinakar et al., 2010a; Vishwakarma et al., 2014; Garmash et al., 2021a), drought (Dahal and Vanlerberghe, 2017; Challabathula et al., 2022), osmotic (Saradadevi and Raghavendra, 1994; Dinakar et al., 2016); temperature (Saradadevi and Raghavendra, 1994; Searle et al., 2011; Dinakar et al., 2016), salt (Analin et al., 2020), normoxia and hypoxia (Jayawardhane et al., 2020). The mETC is known to benefit photosynthesis by the following suggested mechanisms: (i) indirect removal of surplus amount of chloroplastic reducing equivalent by malate-OAA and triose-P-PGA shuttle (Krömer and Scheibe, 1996; Padmasree and Raghavendra, 1999c; Scheibe et al., 2005; Yoshida et al., 2007; Dinakar et al., 2010a; Zhang et al., 2017; Garmash, 2021b) (ii) dampening excess ROS through nonenzymatic and enzymatic ROS scavengers (Dinakar et al., 2010b; Vishwakarma et al., 2015; Dinakar et al., 2016; Cheng et al., 2020; Garmash et al., 2020; Challabathula et al., 2022; Manbir et al., 2022) (iii) expedite photosynthetic induction and light activation of major chloroplastic enzymes (Padmasree and Raghavendra, 1999b, Padmasree and Raghavendra, 2001a; Batnini et al., 2020) and (iv) bestow ATP to the cytosol for the synthesis of sucrose (Padmasree and Raghavendra, 1999c; Igamberdiev et al., 2006).

Several recent studies revealed the involvement of brassinosteroids (BRs) in various physiological processes such as photosynthesis (Jiang et al., 2012a; Jiang et al., 2012b), stress tolerance (Lima and Lobato, 2017; Dos Santos Ribeiro et al., 2019; Fu et al., 2019) and activation of mitochondrial AOX pathway to alleviate photoinhibition (Deng et al., 2015; Wei et al., 2015; Arfan et al., 2019). Apart, BRs, which are known to be ubiquitously distributed in the plant kingdom (Clouse and Sasse, 1998; Bishop and Koncz, 2002; Krishna, 2003; Bajguz and Hayat, 2009; Bhanu, 2019; Peres et al., 2019; Hussain et al., 2020; Raza et al., 2022), play imperative roles in a multitude of physiological function, including the stimulation of a broad spectrum of cellular responses such as cell lengthening (Clouse and Sasse, 1998; Lanza et al., 2012; Liu et al., 2018; Ruan et al., 2018), pollen tube growth (Ye et al., 2010), blossom phase (Domagalska et al., 2010), seed establishment (Jiang et al., 2013a) induction of ethylene biosynthesis (Wei et al., 2015), regulation of expression of the photosynthetic genes (Cheng et al., 2014). BRs are also known to activate adaptive mechanisms in response to diverse environmental stresses, i.e., high temperature (Zhang et al., 2013), water deficit (Lima and Lobato, 2017; Dos Santos Ribeiro et al., 2019; Wang et al., 2022), cold (Fu et al., 2019; Heidari et al., 2021; Fu et al., 2022; Wang et al., 2022), salt (Rattan et al., 2020), drought (Naservafaei et al., 2021), and chromium (Basit et al., 2022). Thus, BRs are suggested as promising plant growth regulators for improving the yield of essential crops (Khripach et al., 2000; Divi and Krishna, 2009; Nolan et al., 2020). Further, the cellular redox environment is reconfigured in the presence of BRs to enhance photosynthetic efficiency (Jiang et al., 2012b). However, so far, no clear information is available on intracellular adjustments of redox and ROS through modulating mETC during the optimization of photosynthesis in response to BRs.

The present study was performed using *A. thaliana* mesophyll cell protoplasts as an experimental prototype to ascertain the significance of BRs along with mETC in mediating the beneficial interactions between chloroplasts and mitochondria during the optimization of photosynthesis by monitoring changes in various biochemical components associated with malate valve and antioxidant systems.

## Materials and methods

### Plant material and growth conditions

The seeds of *Arabidopsis thaliana* L. Heynh ecotype of Columbia (Col-0) were sown on artificial soil (SOILRITE-MIX, Keltech Energies Limited, Bengaluru, India) supplemented with half-strength Murashige and Skoog (MS) medium (Murashige and Skoog, 1962) and kept in the dark for 3 to 4 d at 2 to 4°C for stratification as described in Vishwakarma et al. (2014). Later, the pots were transferred to a growth chamber set at 8h photoperiod and grown under a light intensity of 50-60 µmol m^-^² s^-^¹ at 22°C (Vishwakarma et al., 2015). After 30 days, young plants, which are at the 4 to 6^th^ leaf stage, were transferred to individual pots. The fully grown leaves were collected from 12 to early 14 weeks old plants as it was easy to peel the lower epidermis for isolation of mesophyll cell protoplasts (MCP) and preparation of leaf discs because of the expanded lamina. The leaf discs were made from the leaf blade excluding the midrib using a sharp paper puncher under the water.

### Separation of mesophyll cell protoplasts

MCP was separated from *A. thaliana* leaves with minor modifications to the protocol described by Riazunnisa et al. (2007). Leaf strips of 1cm^2^ in size, without lower epidermis (abaxial) and midrib, were placed in a Petri plate with a peeled surface touching the pre-plasmolysis medium (0.60 M sorbitol, 1mM CaCl_2_, 5mM MES-KOH pH 6.0). After 30 min, the pre-plasmolysis medium was removed. The digestion medium containing 0.65 M sorbitol, 1mM CaCl_2_, 5mM MES-KOH (pH 5.5), 0.25 mM Na_2_EDTA, 5mM sodium ascorbate, 0.2% BSA, 1% Cellulase Onozuka R-10 and 0.4% Macerozyme R-10 (Yakult Honsha Co. Ltd, Nishinomiya, Japan) was added to the leaf strips present in Petri plate and kept at 30°C for 45 min, under the illumination of 50-100 µmol m^-2^s^-1^ to digest the cell wall. After digestion, the digestion medium was discarded, and immediately washing medium (0.65 M sorbitol, 1 mM CaCl_2_, 5 mM MES-KOH pH 6.0) was supplied. MCP was released in the washing medium by gently tapping and swirling the Petri plate. The released MCP was filtered through a nylon membrane (60 µm) to remove the debris. The intact pure MCP was collected using a swing-out head centrifuge. The MCP is delicate and therefore centrifuged at a low speed of 1000 rpm for 5min at 4°C. The centrifugation step was repeated twice/thrice to remove the broken MCP. The pellet was subsequently suspended in a suspension medium (0.65 M sorbitol, 1 mM CaCl_2_, 10 mM HEPES-KOH pH 7.0) and centrifuged to remove the traces of the washing medium. A small aliquot of suspension medium was added to the final pellet, and the MCP suspension was kept on ice to perform all the experiments described below. Neutral red and Evans blue were used to analyze the quality and viability of MCP under the microscope. More than 90% intact protoplasts were used. The Arnon (1949) method for the estimation of chlorophyll (Chl) was used to estimate the amount of Chl.

### Treatment with brassinolide in the presence (or) absence of mitochondrial inhibitor

Initially, MCP have been exposed independently to a wide range of brassinolide (BL) (0.5 pM to 5 pM) concentrations in a pre-incubation chamber (perplex) containing reaction medium (0.65 M sorbitol, 1mM CaCl_2_, 1mM MgCl_2_, 10mM HEPES-KOH pH 7.5) for 10 min in light at 25°C. In later experiments, MCP is pre-incubated with an optimized concentration of BL (0.5 pM) in the presence and absence of AA (100 nM) or SHAM (0.5 mM), respectively under similar conditions described above. During pre-incubation, an illumination of 1000 µmol m^-2^ s^-1^ was provided using LED lamps as this light intensity resulted in maximal photosynthetic rates in MCP of *A. thaliana* (Strodtkötter et al., 2009). Subsequently, the MCP is used either for monitoring photosynthesis, respiration, the capacity of COX or AOX pathway, ROS, enzyme assay (or) stored in liquid nitrogen to quantify total cellular redox ratios of pyridine nucleotide(s), pyruvate, malate, and glucose-6-phosphate (Glc-6-P).

For transcript analysis, leaf discs that were treated with 0.1μM BL in the presence and absence of 20 μM AA or 1mM SHAM were used (Strodtkötter et al., 2009; Deng et al., 2015). All the treatments were given in light (50 µmol m^-2^s^-1^) for 6 hours under DDW containing 0.01% Tween-20 (Vishwakarma et al., 2015) and transferred to liquid N_2_.

### Monitoring photosynthesis and respiration in MCP

An aliquot (10 µg Chl) of MCP after treatment with BL in the presence or absence of mitochondrial inhibitors was transferred to an Oxygraph cuvette comprising of Clark-type oxygen electrode system (Model DW2, Hansatech Ltd., King’s Lynn, UK) to monitor respiration and photosynthesis at 25°C. The total respiration (TR) was determined as O_2_ uptake under dark conditions (for 5 min). Besides, photosynthesis was measured as (i) a NaHCO_3_ (1mM) dependent O_2_ evolution which represents photosynthetic carbon assimilation (PCA) or Calvin-Benson cycle (CBC) activity, and (ii) *para*-Benzoquinone (1mM) dependent O_2_ evolution which represents PSII activity, under the optimal light intensity of 1000 µmol m^-2^s^-1^ for 10 min (Padmasree and Raghavendra, 2001b; Strodtkötter et al., 2009). NaHCO_3_ or *para*-Benzoquinone is added to the reaction medium soon after switching on a light.

### Evaluation of the different components of TR in MCP

The various components of TR, i.e., the capacity of the COX pathway, AOX pathway, and residual respiration (RR), were assessed in MCP, as explained by Dinakar et al. (2010a). Thus, the capacity of the AOX pathway was examined as the O_2_ uptake sensitive to 10 mM SHAM in the presence of 1 mM KCN. On the other hand, the capacity of the COX pathway was analyzed as O_2_ uptake sensitive to 1mM KCN in the co-existence of 10mM SHAM and 1µM CCCP (Carbonyl cyanide-3-chlorophenylhydrazone). CCCP was used to ensure that adenylate control does not limit respiration (McDonald et al., 2002).

### Assessment of reduced and oxidized forms of pyridine nucleotide in MCP

MCP corresponding to 25 µg Chl was subjected to various treatments and quickly stored in cryogenic nitrogen. Later, samples were liquefied and spun at a centrifugal force of 3000 g for 2 min. Further, 0.2 N HCl and 0.2 M NaOH were treated with pelleted MCP to analyze NAD(P)^+^ and NAD(P)H, respectively. The homogenized extract was centrifuged at 10,000 *g* for 10 min at 4°C (Dinakar et al., 2016). The supernatant was heated at 100 °C for 1 min, followed by cooling on ice. The pH of the supernatant was modified for the quantification of NAD(P)^+^ (pH 5 to 6) and NAD(P)H (pH 7 to 8). Further, NAD(H) levels were assessed by ethanol consumption with the help of alcohol dehydrogenase, which in turn coupled with phenazine methosulfate-dependent reduction of dichlorophenolindophenol. In contrast, the NADP(H) amounts were quantified by utilization of glucose-6-phosphate (Glc-6-P) by Glc-6-P dehydrogenase, which in turn linked with phenazine methosulfate-dependent reduction of dichlorophenolindophenol. Declined absorbance at 600 nm was examined for 3 min. The amount of pyridine nucleotides was determined by respective standards of NAD(P)^+^ and NAD(P)H (Queval and Noctor, 2007).

### Evaluation of reactive oxygen species in MCP

MCP was loaded with 5µM fluorescent dye 2,7-dichlorofluorescein diacetate (H_2_DCFDA) to monitor the intracellular production of ROS as described in Dinakar et al. (2010b). After incubation, MCP was treated with or without BL in the presence or absence of AA or SHAM for 10 min in a preincubation chamber under a photon flux density of 1000 μmol m^-2^ s^-1^ at 25 °C. Subsequently, the treated MCP was used to quantify DCF fluorescence using a spectrofluorometer (FP-8500, Jasco, Japan) run at excitation and emission wavelengths of 488 and 525 nm, respectively. Further, fluorescence generated due to ROS formation in MCP was observed under a Laser scanning confocal microscope (LSM 710, Carl Zeiss, Oberkochen, Germany) with excitation and an emission filter of 488 nm and 525 nm, respectively.

### Quantification of pyruvate, malate, and Glc-6-P in MCP

The MCP equal to 100 µg Chl contained in 600 µl reaction medium subjected to various treatments was treated with HClO_4_ (final concentration of 3%) and instantly frozen in liquid nitrogen as described in Padmasree and Raghavendra (1999a, 1999b). Later, the thawed samples were neutralized with KOH and immediately spun at 7000 g. Subsequently, the supernatant was chosen to assess the total cellular level of pyruvate, malate, and Glc-6-P using a UV-Visible spectrophotometer (Shimadzu-1700 PharmaSpec UV-Vis). The pyruvate levels were measured using an enzymatic assay coupled with the utilization of NADH. In contrast, Glc-6-P levels were measured by an enzymatic assay associated with the formation of NADPH. In contrast, the malate is quantified as an enzymatic assay linked with the generation of NADH.

### Enzyme assays in MCP

MCP equivalent to 1 to 5 µg Chl in the reaction medium was subjected to various treatments and immediately used for enzyme assays. The maximal and actual enzymatic activities of the following light-activated chloroplastic enzymes of CBC: NADP-glyceraldehyde-3-phosphate dehydrogenase [NADP-GAPDH (EC 1.2.1.13)], fructose-1,6-bisphosphatase [FBPase (EC 3.1.3.11)] and phosphoribulokinase [PRK (EC 2.7.1.19)] was quantified spectrophotometrically (Shimadzu-1700 PharmaSpec UV-Vis). Protoplasts were added to the sample and reference cuvette during the assays. In contrast, the substrate(s) were added to only the sample cuvette according to the protocol described in Padamsree and Raghavendra (2001a). Triton X-100 [0.02% (v/v)] was included in the assay medium to solubilize the protoplasts. The activity of FBPase was assayed by monitoring the NADP reduction (increase in A340), while the activity of NADP-GAPDH was assayed by monitoring the NADPH oxidation (decrease in A340). However, the PRK activity is quantified by NADH oxidation (decline in A340). The maximal enzymatic activity of these three enzymes was determined after adding 50 mM DTT to the reaction medium containing protoplast in light.

### Total RNA isolation and cDNA synthesis from leaf discs

Total RNA was extracted from 100 mg of leaf tissue from different treatments using TRI Reagent (Sigma Aldrich, USA) according to the manufacturer’s instructions. The total RNA and its quality in each sample were analyzed using a Nano-Drop ND-2000c spectrophotometer (Thermo-Fisher Scientific). One µg of total RNA with an A_260_/A_280_ ratio between 1.9 to 2.0 and an A_260_/A_230_ ratio between 2.0 to 2.2 was used for first-strand cDNA synthesis using Prime Script ™ 1^st^ strand cDNA Synthesis kit (Takara Bio Inc., Shiga, Japan) according to the instructions given by the manufacturer.

### Real-time PCR

The PCR sample comprised 2 µl of diluted cDNA, 5 µl of TB Green Premix Ex Taq™ II Master Mix (Takara Bio Inc., Shiga, Japan), 0.8 µl of ROX dye and 1µl (0.1pM) of specific primers in a final volume of 10 µl. Real-time PCR was carried out after preincubation at 95°C for 10 min followed by 40 cycles comprised of denaturation at 95°C for 15 s and annealing/extension at 60°C each for 1 min using Step One plus real-time PCR machine (Applied Biosystems, USA) as described by Vishwakarma et al. (2014). Cycle threshold (C_T_) values were deduced from the log phase of PCR amplification. The results were evaluated using the comparative C_T_ method (Livak and Schmittgen, 2001). This procedure produced a C_T_ value (ΔC_T_ = GOIC_T_-*UBQ5*C_T_) by comparing the expression of a gene of interest (GOI) to that of *UBQ5* (a reference gene). Equation 2^-ΔΔC_T_^ were then used to quantify the transcript abundance.

### Real-time PCR primers

Real-time PCR primers for the genes ubiquitin (*UBQ5*) as a reference, chloroplastic-malate dehydrogenase (*Chl-MDH*), mitochondrial malate dehydrogenase (*M-MDH*), peroxisomal/chloroplastic catalase (*CAT1*), cytosolic superoxide dismutase (*CSD1*), thylakoid ascorbate peroxidase (*tAPX*), stromal ascorbate peroxidase (*sAPX*) were same as described in Vishwakarma et al. (2014). In contrast, the primer for the following genes: glutathione reductase (*GR*), monodehydroascorbate reductase (*MDHAR*), dehydroascorbate reductase (*DHAR*), glyceraldehyde-3-phosphate dehydrogenase (*GAPDH*), fructose-1,6- bisphosphatase (*FBPase*) and phosphoribulokinase (*PRK*) was designed using Primer-Blast, NCBI, NIH, Bethesda, Maryland, USA. Before primer blasts, the 5’UTR sequence of the respective gene was retrieved from transcriptomic sequence using Phytozome, the plant comparative genomic portal of the Department of Energy Joint Genome Institute, University of California and Gene Structure and Display Server (GSDS), Centre for Bioinformatics, Peking University. The sequence of forward and reverse primers for all the genes examined in the present study is shown in the **Supplementary Table 1**.

### Statistical analysis

The experimental data depicted in the present study is an average of the observations from three studies carried out on different days. One-way ANOVA was used to compare the variation among treatments. The Tukey's test of multiple comparison analysis was performed using the Sigma Plot 14.0 program (San Jose, CA, USA).

## Results

### TR and PCA of MCP pre-incubated under a series of BL concentration

**Figure 1:**
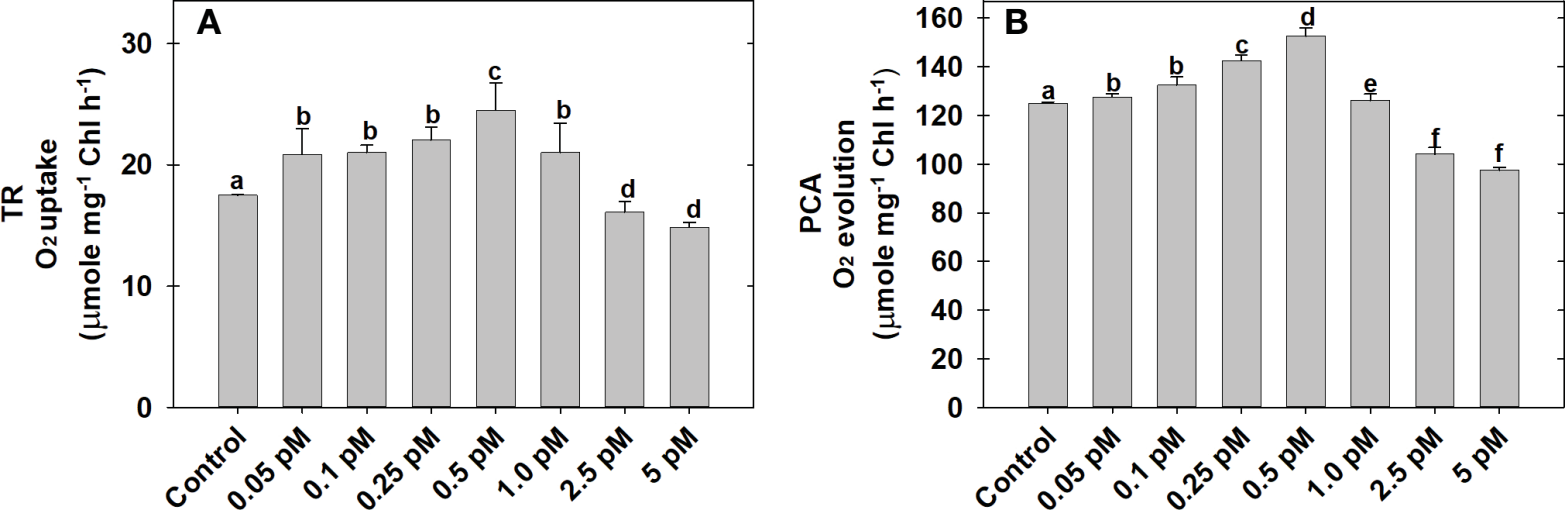
Effect of different concentrations of BL on TR **(A)** and PCA **(B)** in MCP of *A*. *thaliana*. The MCP in the reaction medium was pre-incubated with a series of BL concentrations (0.05 to 5 pM) for 10 min under a light intensity of 1000 μmol m^-2^ s^-1^ at 25°C. After treatment, the TR was recorded as O_2_ uptake for 5 minutes in the dark and PCA was traced as NaHCO_3_-dependent (1.0 mM) O_2_ evolution for 10 min in light (1000 μmol m^-2^ s^-1^) using a Clark-type oxygen electrode. More detailed information on the procedure is mentioned in the “materials and methods” section. A statistically significant difference is represented by different lowercase alphabetical letters (P < 0.05) and values represent the average (± SE) of three experiments.

The effect of BL regimes on TR as O_2_ uptake and PCA as bicarbonate-dependent O_2_ evolution is determined using MCP as the experimental system (**Figures 1A, B**). Among the series of BL concentrations (0.05 to 5.0 pM) used in the present study, the maximum rates of O_2_ uptake (24.5 ± 0.5 μmol mg^−1^Chl h^−1^) and O_2_ evolution (158.5 ± 1.43 μmol mg^−1^Chl h^−1^) was observed at 0.5 pM BL as compared to the rates of O_2_ uptake (17.5 ± 0.8 μmol mg^−1^Chl h^−1^) and O_2_ evolution (125 ± 2.5 μmol mg^−1^Chl h^−1^) without BL under optimal conditions of light and temperature (**Figures 1A, B**).

### Effect of BL on the different components of TR in MCP

**Figure 2:**
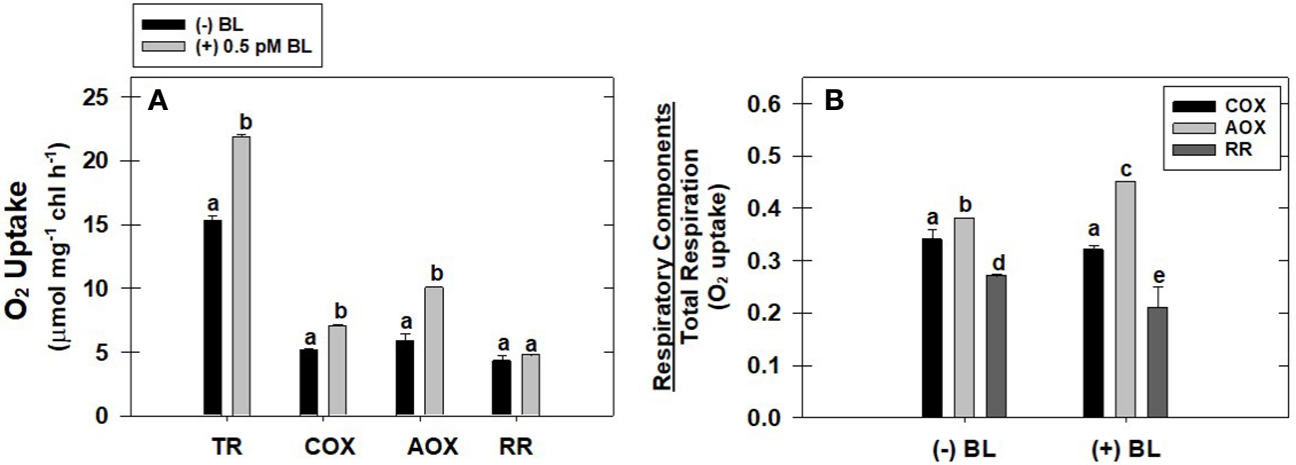
Effect of BL on the different components of TR: capacity of COX pathway, AOX pathway, and RR **(A)**. The ratio of O_2_ uptake by each of these components to total respiration in MCP of *A. thaliana* **(B)**. The MCP in the reaction medium was pre-incubated for 10 min in the presence of 0.5 pM BL at a light intensity of 1000 μmol m^-2^ s^-1^ at 25°C. After treatment, MCP was shifted to an oxygraph cuvette to monitor various components of respiration. Further, the section “materials and methods” mentions more detailed information on the procedure. A statistically significant difference is represented by different lowercase alphabetical letters (P ≤ 0.05) and values represent the average (± SE) of three experiments.

The effect of optimal concentration of BL (0.5 pM) on the capacity of the COX pathway, AOX pathway, and residual respiration (RR) was evaluated with reference to TR in MCP after pre-incubation under light (**Figure 2A**). BL enhanced the O_2_ uptake of the COX pathway by 36%, the AOX pathway by 81%, and residual respiration by 13% when compared with their O_2_ uptake rates in the absence of BL (**Figure 2A**). However, the ratio of AOX pathway capacity to TR is significantly enhanced upon treatment with BL from 0.38 ± 0.01 (without BL) to 0.45 ± 0.016. In contrast, the addition of BL decreased the ratio of COX pathway capacity to TR from 0.34 ± 0.02 (without BL) to 0.32 ± 0.02 and the ratio of RR to TR from 0.27± 0.021 (without BL) to 0.21± 0.011 (**Figure 2B**).

### Effect of BL on TR, PCA, and PSII in the presence of mitochondrial inhibitors

**Figure 3:**
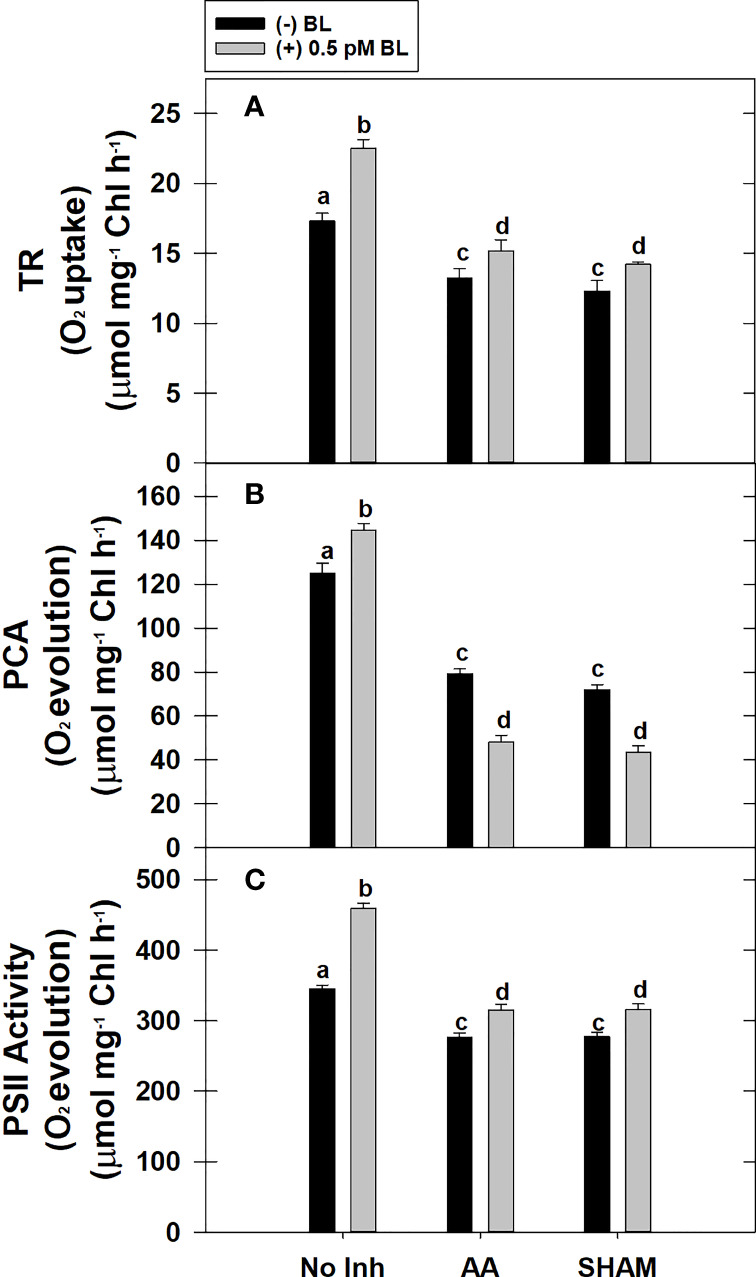
Effect of BL on TR **(A)**, PCA **(B)** and PS II activity **(C)** under the disruption of COX pathway or AOX pathway in MCP of *A*. *thaliana*. The MCP in the reaction medium was pre-incubated with or without 0.5 pM BL in the presence or absence of 100nM AA (COX pathway Inhibitor) or 0.5 mM SHAM (AOX pathway inhibitor) for 10 min at a light intensity of 1000 μmol m^-2^ s^-1^ at 25°C. After treatment, MCP was shifted to an oxygraph cuvette to monitor respiration in the dark and photosynthesis under the light. Further, the section “materials and methods” mentions more detailed information on the procedure. No Inhibitor (No Inh) depicts a sample without mETC inhibitors. A statistically significant difference is represented by different lowercase alphabetical letters (P ≤ 0.05) and values represent the average (± SE) of three experiments.

The TR of MCP was enhanced by 30% after pre-incubation with 0.5 pM BL in light when compared with TR in the absence of BL in light. But, when the COX or AOX pathway of mETC is partially disrupted either with AA (or) SHAM, BL could not enhance the TR to the extent observed in the absence of mitochondrial inhibitors. Thus, adding BL in the presence of AA or SHAM could enhance the TR by ~15% of their control rates, i.e., observed in the presence of AA and SHAM alone (**Figure 3A**).

The PCA rate of MCP was significantly enhanced by 27% upon adding BL in light. However, adding BL to MCP after disrupting mETC with either AA or SHAM could not stimulate the PCA. The decrease in rates of PCA was further aggravated by ~40% when BL was added to MCP containing AA or SHAM when compared with rates of PCA that were decreased in the presence of AA (37%) or SHAM (42%) alone (**Figure 3B**).

Similar to PCA, the PSII activity of MCP was significantly enhanced by 33% upon treatment with BL in light. But, BL could not stimulate the PSII activity to this extent when mETC is disrupted with either AA or SHAM. However, BL enhanced the PSII rates in the presence of AA or SHAM by ~14% as compared to the PSII rates that were decreased with the addition of AA or SHAM alone (**Figure 3C**).

### Effect of BL on the activity of CBC enzymes in the presence of mitochondrial inhibitors

**Figure 4:**
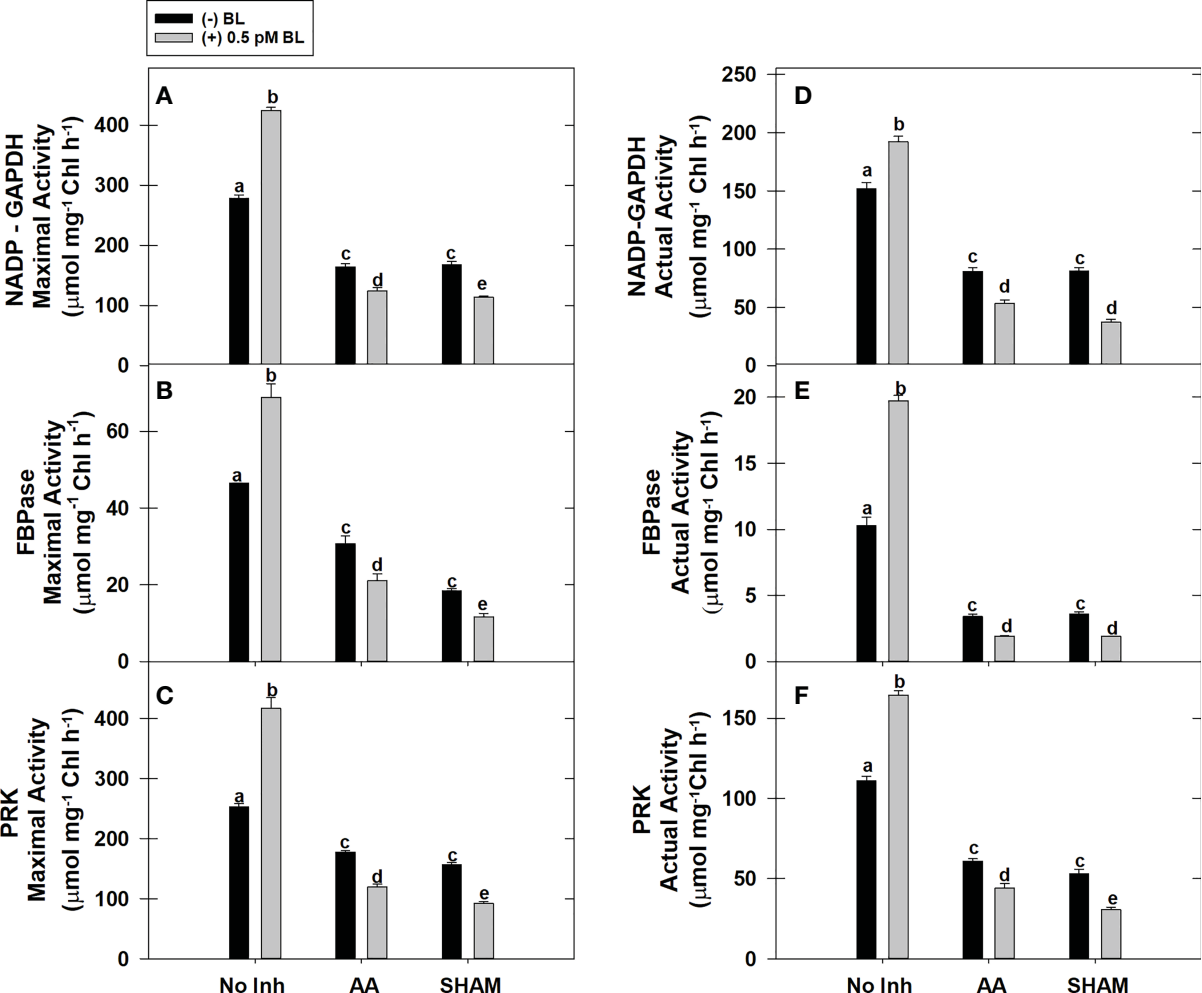
Effect of BL on maximal activity **(A–C)** and actual enzymatic activity **(D–F)** of NADP-GAPDH, Stromal FBPase, and PRK under the disruption of COX pathway or AOX pathway in MCP of *A. thaliana*. The MCP in the reaction medium was pre-incubated with or without 0.5 pM BL in the presence or absence of 100nM AA (COX pathway Inhibitor) or 0.5 mM SHAM (AOX pathway inhibitor) for 10 min at a light intensity of 1000 μmol m^-2^ s^-1^ at 25°C. After treatment, MCP was immediately used to monitor maximal and actual activity. Further, the section “materials and methods” mentions more detailed information on the procedure. No Inhibitor (No Inh) depicts a sample without mETC inhibitors. A statistically significant difference is represented by different lowercase alphabetical letters (P ≤ 0.05) and values represent the average (± SE) of three experiments.

The actual activity (≤65%) and the maximal activity (≤91%) of redox-sensitive CBC enzymes NADP-GAPDH, PRK, and FBPase were increased significantly in MCP upon the addition of BL in light (**Figures 4A–F**). But, both of these activities could not be promoted in MCP by BL when the mETC is disrupted by either AA or SHAM, as observed in samples without mitochondrial inhibitors. Thus, the addition of BL to MCP containing AA (≤43%) or SHAM (≤55%) further aggravated the decreased pattern in the enzymatic activities (maximal and actual) of all the enzymes that were observed with the addition of AA or SHAM alone (**Figures 4A–F**).

### Effect of BL on Glc-6-P, pyruvate, and malate in the presence of mitochondrial inhibitors

**Figure 5:**
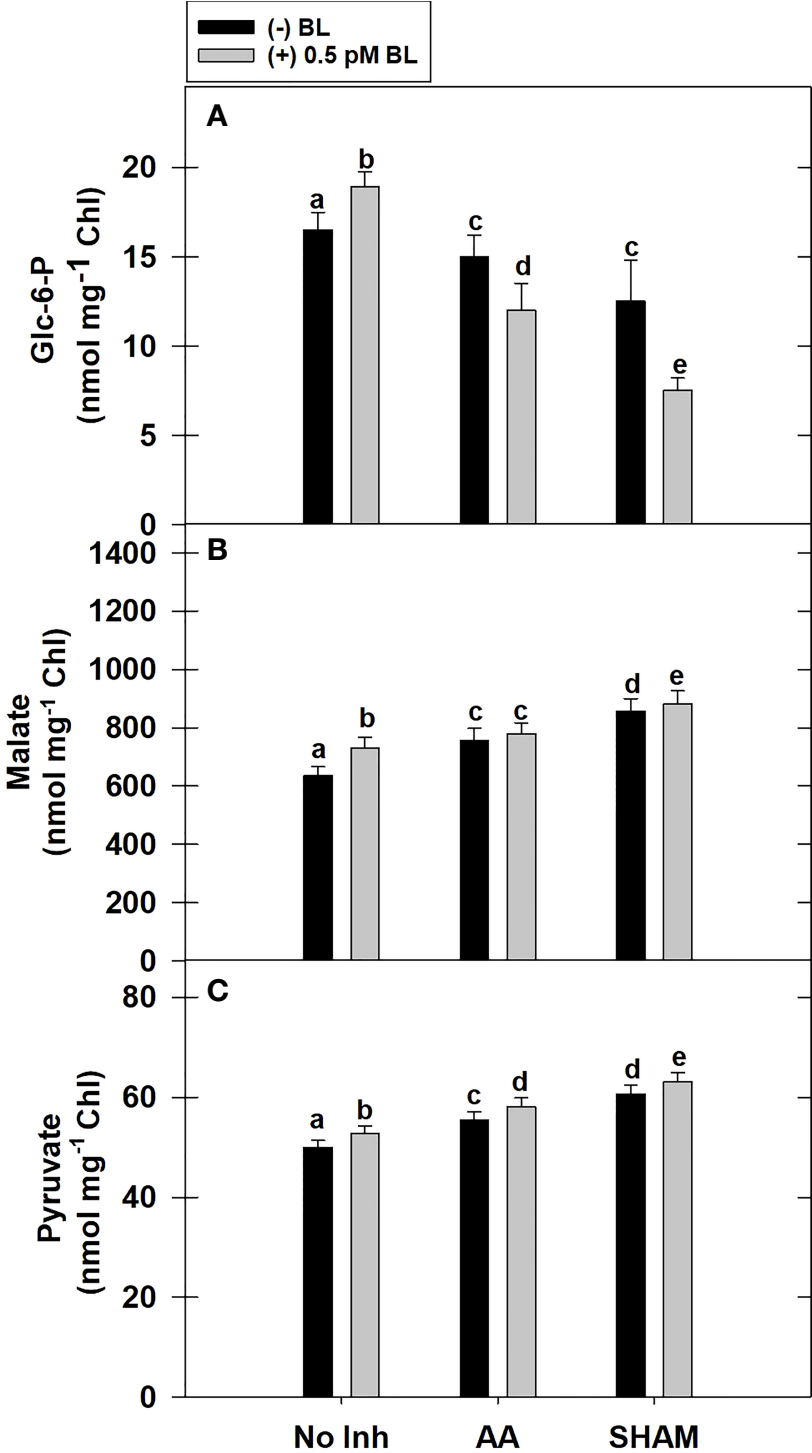
Effect of BL on the total cellular level of Glc-6-P **(A)**, Malate **(B)** and Pyruvate **(C)** under the disruption of COX or AOX pathway in MCP of *A*. *thaliana*. The MCP in the reaction medium was pre-incubated with or without 0.5 pM BL in the presence or absence of 100nM AA (COX pathway Inhibitor) or 0.5 mM SHAM (AOX pathway inhibitor) for 10 min at a light intensity of 1000 μmol m^-2^ s^-1^ at 25°C. After treatment, MCP was immediately quenched by HClO_4_ and stored in liquid N_2_ for analysis. Further, the section “materials and methods” mentions more detailed information on the procedure. No Inhibitor (No Inh) depicts a sample without mETC inhibitors. A statistically significant difference is represented by different lowercase alphabetical letters. (P ≤ 0.05) and values represent the average (± SE) of three experiments.

Adding BL to MCP has remarkably increased the total cellular levels of Glc-6-P by ~15% in light. Similar to PCA observed above, the addition of BL decreased the cellular Glc-6-P levels when mETC was disrupted by either AA or SHAM. In fact, the decrease in Glc-6-P levels was further aggravated when BL was added to MCP containing AA (20%) or SHAM (40%) when compared with the Glc-6-P levels of MCP that were decreased with the addition of AA (<10%) or SHAM (26%) alone (**Figure 5A**).

Similar to Glc-6-P, the total cellular levels of malate were increased remarkably by ~15% with the addition of BL to MCP in light. The addition of BL to MCP containing either AA or SHAM further enhanced the cellular malate levels. But, the rise in malate levels was marginal (<4%) when BL was added to MCP containing AA or SHAM when compared with the increase in malate levels of MCP that were treated with AA (19%) or SHAM alone (34%) (**Figure 5B**).

The total cellular levels of pyruvate increased marginally by <5% when BL was added to MCP in light. Also, adding BL to MCP containing AA or SHAM further raised the pyruvate levels. But the rise in pyruvate levels was marginal (<5%) when BL was added to MCP having either AA or SHAM when compared with the pyruvate levels of MCP treated with AA (11%) or SHAM (20%) alone (**Figure 5C**).

### Effect of BL on redox ratio of pyridine nucleotides in the presence of mitochondrial inhibitors

**Figure 6:**
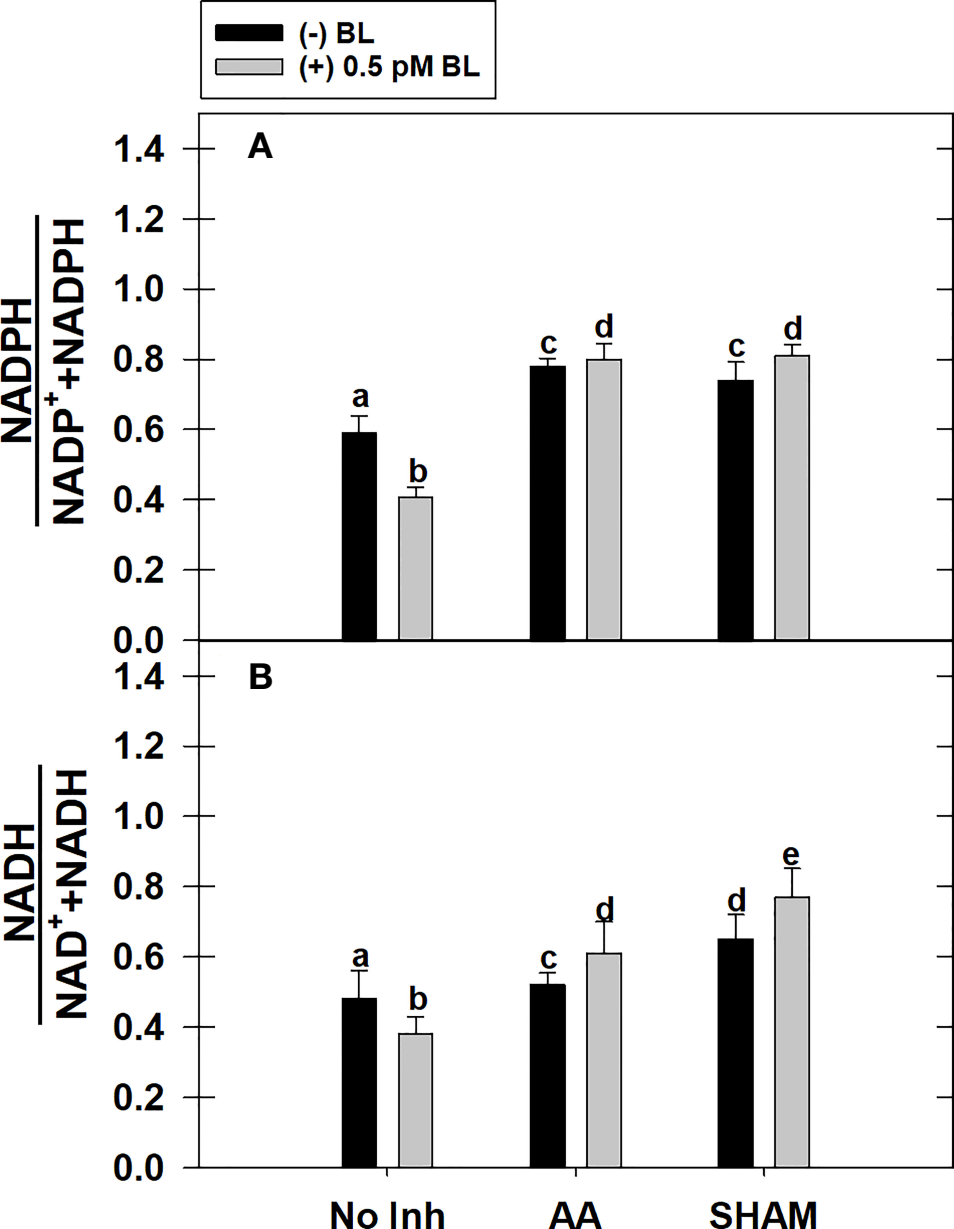
Effect of BL on the total cellular level of redox ratio related to NADP(H) **(A)** and NAD(H) **(B)** under the disruption of COX pathway or AOX pathway in MCP of *A*. *thaliana*. The MCP in the reaction medium was pre-incubated with or without 0.5 pM BL in the presence or absence of 100nM AA (COX pathway Inhibitor) or 0.5 mM SHAM (AOX pathway inhibitor) for 10 min at a light intensity of 1000 μmol m^-2^ s^-1^ at 25°C. After treatment, MCP was immediately stored in liquid N_2_ for analysis. Further, the section “materials and methods” mentions more detailed information on the procedure. No Inhibitor (No Inh) depicts a sample without mETC inhibitors. A statistically significant difference is represented by different lowercase alphabetical letters (P ≤ 0.05) and values represent the average (± SE) of three experiments.

Adding BL to MCP significantly decreased the cellular redox ratio of (i) NADPH to NADP^+^ and NADPH and (ii) NADH to NAD^+^ and NADH by 31% and 21%, respectively, in light. But, this effect of BL on redox ratios related to NADPH or NADH was reversed when it was added to MCP containing either AA or SHAM. However, the rise in redox ratio related to NADPH upon adding BL to MCP having either AA or SHAM did not vary with those samples of MCP containing AA (32%) or SHAM (26%) alone. In contrast, the rise in redox ratio related to NADH was further aggravated upon the addition of BL to MCP containing either AA (17%) or SHAM (18%) than that of MCP (without BL) where redox ratio of NADH was enhanced by 8% in the presence of AA and 35% in the presence of SHAM, respectively (**Figures 6A, B**).

### Effect of BL on cellular ROS in the presence of mitochondrial inhibitors

**Figure 7:**
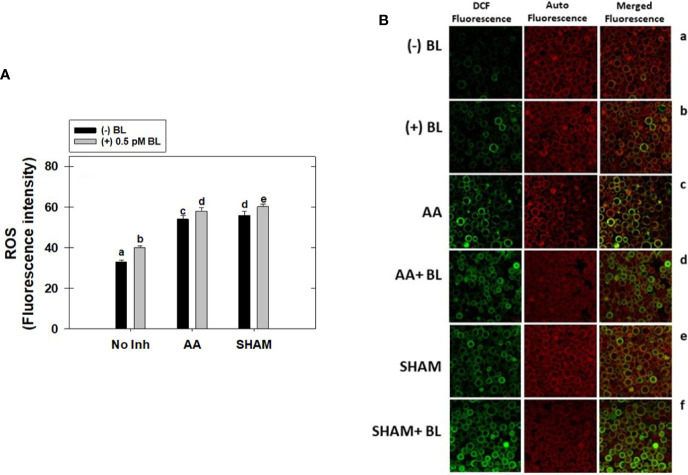
Effect of BL on total cellular ROS **(A)** and Confocal Microscopic images of ROS **(B)** under the disruption of COX pathway or AOX pathway in MCP of *A*. *thaliana*. The MCP in the reaction medium was pre-incubated with or without 0.5 pM BL in the presence or absence of 100nM AA (COX pathway Inhibitor) or 0.5 mM SHAM (AOX pathway inhibitor) for 10 min at a light intensity of 1000 μmol m^-2^ s^-1^ at 25°C. Further, the section “materials and methods” mentions more detailed information on the procedure. No Inhibitor (No Inh) depicts a sample without mETC inhibitors. A statistically significant difference is represented by different lowercase alphabetical letters (P ≤ 0.05) and values represent the average (± SE) of three experiments.

Adding BL to MCP significantly raised the total cellular ROS levels by 21% in light. This increase in ROS was further enhanced when mETC was disrupted with mitochondrial inhibitors. But, the rise in ROS was marginal (<7%) upon the addition of BL to MCP containing either AA or SHAM when compared with their respective control samples where MCP had AA (63%) or SHAM (69%) alone (**Figure 7A**).

The cellular ROS of MCP upon treatment with BL in the presence and absence of mitochondrial inhibitors was further confirmed by confocal microscopic studies using H_2_DCF-DA (**Figure 7B**). The ROS in the chloroplastic region was shown by a pseudo-orange/yellow color formed due to the superimposition of chlorophyll autofluorescence and DCF fluorescence, and the ROS in the extra-chloroplastic region is visible as DCF fluorescence. In the control (MCP without BL or mitochondrial inhibitors), a marginal level of DCF fluorescence was observed. At the same time, adding BL enhanced fluorescence in both chloroplastic and extra-chloroplastic regions (**Figure 7A, a, b**). This increase in both chloroplastic and extra-chloroplastic fluorescence was further aggravated when BL was added to MCP containing mitochondrial inhibitors when compared with fluorescence in MCP with AA or SHAM alone (**Figure 7B, c–f**).

### Effect of BL on the transcript levels of important genes associated with CBC enzymes, malate valve, and antioxidant system in the presence of mitochondrial inhibitors

**Figure 8:**
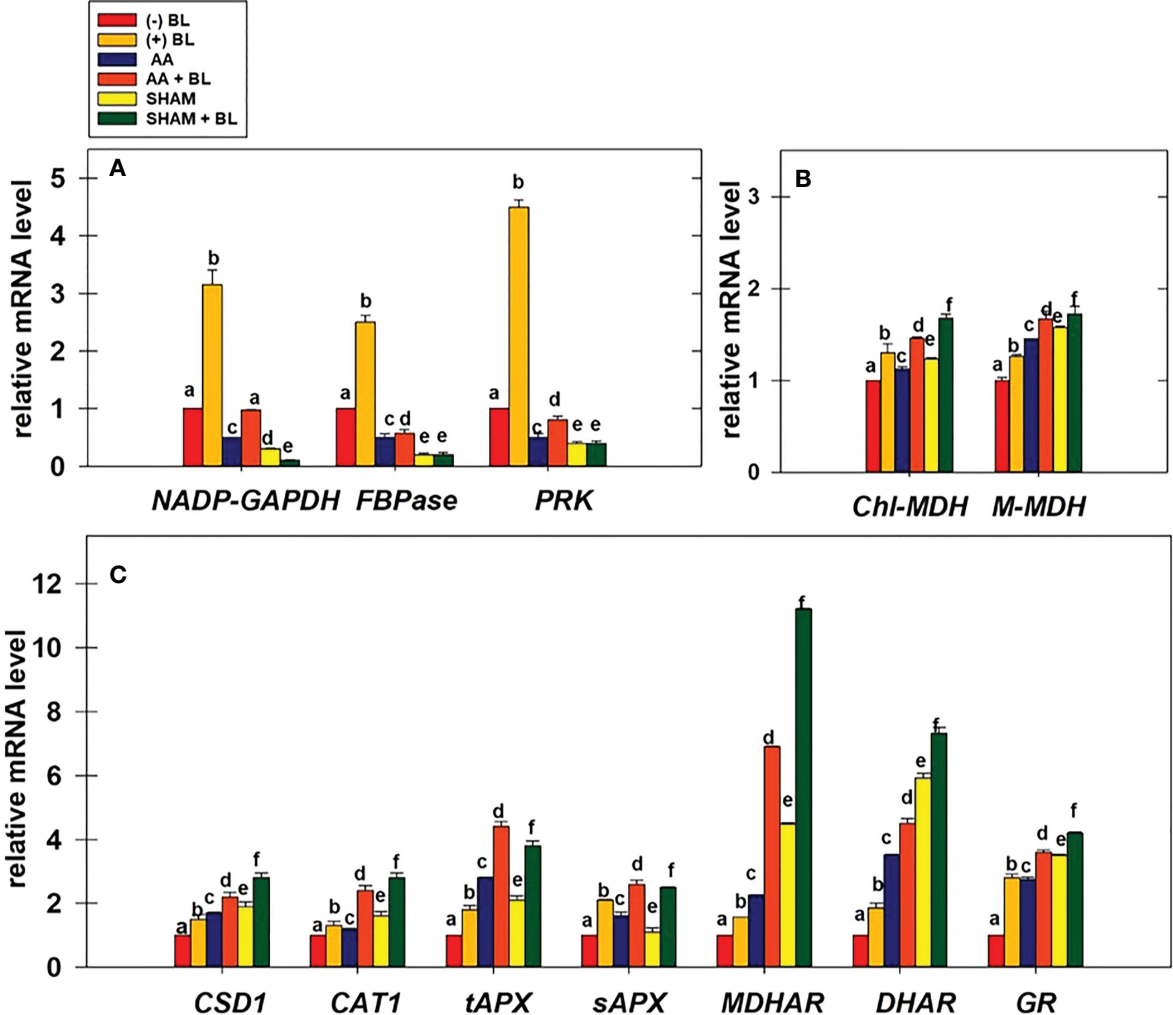
Effect of BL on gene expression profiles of enzymes related to CBC: *NADP-GAPDH*, *FBPase*, and *PRK* **(A)**; malate valve: *Chl-MDH*, *M-MDH* **(B)**; antioxidant system: *CSD1*, *CAT1, tAPX*, *sAPX*, *MDHAR*, *DHAR*, and *GR* **(C)** under the disruption of COX pathway or AOX pathway. Total mRNA was isolated after treatment of leaf discs with or without BL (0.1 μM) in the presence or absence of AA (20 μM) or SHAM (0.5 mM) for 6 hours at a light intensity of 50 µmol m^-2^ s^-1^ at 25 °C. The amount of transcript was quantified by ΔΔC_T_ using *UBQ5* as a positive control. Further, the section “materials and methods” mentions more detailed information on the procedure. No Inhibitor (No Inh) depicts a sample without mETC inhibitors. A statistically significant difference is represented by different lowercase alphabetical letters (P ≤ 0.05) and values represent the average (± SE) of three experiments.

The relative changes in the transcript levels of genes related to redox-sensitive CBC enzymes, malate valve, and antioxidant system were examined in leaf discs under the light in the presence of BL under the restriction of either COX or AOX pathway (**Figures 8A–C**).

Adding BL to leaf discs has significantly increased the expression of CBC enzymes *NADP-GAPDH*, *FBPase*, and *PRK* by ≤3.5-fold in light (**Figure 8A**). But, when BL was added to the leaf discs in the presence of AA or SHAM, the expression of these genes was enhanced but not to the extent observed in leaf discs without mitochondrial inhibitors. Thus, the expression of CBC enzymes increased from 0.5 (without BL) to ≤0.97 (with BL) in leaf discs when mETC is inhibited in the presence of AA. In contrast, with the addition of BL to leaf discs containing SHAM, the expression of CBC enzymes either remained the same or decreased as compared to leaf discs treated with SHAM (<0.4) alone (**Figure 8A**).

The expression of genes related to malate valve (*Chl-MDH* and *M-MDH*) was enhanced significantly by ≤26% when BL was added to leaf discs in light. The expression of these genes was further promoted when BL was added to leaf discs treated with either AA (≤67%) or SHAM (≤72%) as compared to their expression in leaf discs treated with AA (≤45%) or SHAM (≤57%) alone (**Figure 8B**).

The expression of genes related to ROS scavenging anti-oxidant system (*CSD1*, *CAT1*, *tAPX*, *sAPX*, *MDHAR*, *DHAR*, and *GR*) was significantly increased by ≤ 2.8-fold in the presence of BL. The addition of BL to MCP containing AA (≤6.9 fold) or SHAM (≤11.2 fold) further enhanced the expression of these genes when compared with the expression of genes in MCP with AA (≤3.5 fold) or SHAM (≤5.9 fold) alone (**Figures 8C**).

## Discussion

In higher plants, both photosynthesis and respiration form an integral component of plant growth as the former metabolic process is involved in the net gain of biomass while the later metabolic process helps in the release of energy and turnover of carbon skeletons derived from amino- and fatty acids in rapidly growing tissues (Raghavendra et al., 1992). Apart, the respiratory rate is known to increase after long hours of illumination in leaves as well as after short periods (5 to 10 min) of illumination in MCP, called ‘light-enhanced dark respiration’ (Azcón-Bieto and Osmond, 1983; Vani et al., 1990; Reddy et al., 1991). Besides such long term and short term beneficial effects of photosynthesis in promoting mitochondrial respiration, the crucial roles of mETC in optimizing photosynthesis under normal light as well as under various environmental conditions is well established using the system of MCP due to the following benefits over the usage of leaf discs or whole plants: (i) absence of cellulose cell wall and intercellular spaces facilitate the rapid exchange of gases (O_2_ and CO_2_); (ii) minimize the artifact associated with stomatal patchiness during gas exchange; (iii) allow the metabolic process especially the respiration and photosynthesis (PSI, PSII and PCA) to get monitored quickly at low dosage of the exogenous chemical such as metabolic inhibitor, activator or a phytohormone and (iv) ease in imaging ROS and sub-cellular localization of target molecule (Padmasree and Raghavendra, 1999a; Padmasree and Raghavendra, 1999b; Padmasree and Raghavendra, 1999c; Padmasree and Raghavendra, 2001b; Strodtkötter et al., 2009; Dinakar et al., 2010a; Dinakar et al., 2010b; Selinski et al., 2014; Nie et al., 2015; Yan et al., 2015; Dinakar et al., 2016; Sunil et al., 2020).

In the present study, the MCP was treated with a light intensity of 1000 µmol m^−2^ s ^−1^ during both pre-incubation and monitoring of photosynthesis (Calvin-Benson cycle and PSII) as it was found to be optimal intensity for achieving maximal photosynthetic rates (data not shown). Also, the studies of Strodtkötter et al. (2009) monitored the photosynthesis rates of MCP from *A. thaliana* at 1000 µmol m^−2^ s ^−1^. Besides, the MCP isolated from *Pisum sativum* showed steady photosynthetic rates for up to 30 min. Similarly, the MCP from *A. thaliana* also showed stable photosynthetic rates up to 30 to 40 min (data not shown). Therefore, we assume that MCP was active during the entire duration (25 min) of the assay conducted in the present study which includes the time required for pre-incubation under light (10 min) and, monitoring of respiration (5 min) and photosynthesis (10 min). Further, it is easy to perform transcript analysis using leaf discs as compared to MCP due to either one or more of the following reasons: (i) oxygraph chamber or pre-incubation chamber cannot be autoclaved as it is mostly made from acrylic material; (ii) consume a large volume of nuclease-free and sterile water to prepare various media used during preparation of MCP and (iii) require leaf tissue in abundance to prepare MCP. Furthermore, despite treating MCP and leaf discs with different light intensities, the transcript levels, as well as maximal/actual activities of CBC enzymes, exhibited a more or less similar trend upon treatment with BL in the presence of mitochondrial inhibitors. Therefore, transcript data obtained from the leaf discs perhaps could be clubbed with the other parameters analyzed in MCP to decipher the role of mETC in BL-promoted photosynthesis.

Several recent studies showed that BRs influence the interaction between chloroplasts and mitochondria to protect the photosynthetic electron transport chain from photo-inhibition by up-regulating the AOX pathway during salt and drought stress (Wei et al., 2015; Hu et al., 2019). Though the influence of BRs in promoting PCA is known, it is not clear if BR has any role along with mETC in optimizing PCA under normal growth conditions. Thus, the present study revealed the significance of BL in optimizing PCA along with mETC by treating MCP with specific metabolic inhibitors AA (inhibitor of COX pathway) and SHAM (inhibitor of AOX pathway) in light.

### BL promoted both photosynthesis and respiration in MCP

BRs are a set of polyhydroxy steroidal phytohormones. Plant tissues contained BRs in trace quantities i.e., 0.01-100 ng g^-1^ of fresh weight, which is far less than any other phytohormone or metabolite present in it (Clouse, 2004; Xin et al., 2013a; Tarkowská et al., 2016). Further, its level varied according to plant organ type, tissue age, and species (Clouse, 2004). Moreover, the content of BL was not detected even in 1 gm rosette tissue of *A. thaliana* using high-end techniques such as UPLC-MRM3-MS and UPLC-MRM-MS (Xin et al., 2013b). Among the seventy identified BRs, Brassinolide (BL), 24-epibrassinolide (EBL), and 28-homobrassinolide (HBL) are the important biologically active forms and their external application is used in various experimental studies (Peres et al., 2019; Tanveer et al., 2019; Raza et al., 2022). However, all other forms of BRs are either biosynthetic precursors or metabolic products of BL (Clouse, 2004). Therefore, in the present study, BL is used to treat the MCP. In general, phytohormones are effective at a very minimal concentration in reprogramming various physiological processes in plants (Siddiqui et al., 2018). Exogenous application of BRs showed a dose-dependent effect on the growth of plants, where they promoted growth at lower concentrations and retarded growth at higher concentrations (Chaiwanon and Wang, 2015; Nolan et al., 2020). The studies of Jiang et al. (2012b) noted that a moderate concentration of BRs increased the efficiency of PCA. Similarly, in the current study, BL enhanced both TR and PCA in MCP when its concentration was increased from 0.05 to 0.5 pM but decreased when its concentration was increased further from 1.0 to 5.0 pM under optimal conditions of light (1000 μmoles m^-2^ s^-1^) and temperature (25°C) (**Figures 1A, B**). Therefore, 0.5 pM of BL was considered the optimal concentration where both TR and PCA were found to be at maximal rates. This concentration of BL is chosen to treat MCP in all the rest of the experiments performed in the present study.

Mitochondria are known to perform a vital role as a sensor for external stimuli (such as BRs) and can trigger elaborative cellular responses for acclimatization (Berkowitz et al., 2016). In previous reports, plants treated with BRs significantly increased the TR under both control (Deng et al., 2015; Derevyanchuk et al., 2015; Zhu et al., 2016; Arfan et al., 2019) and stress conditions such as salt stress (Derevyanchuk et al., 2017) and cold stress (Arfan et al., 2019). The studies of Derevyanchuk et al. (2015) suggested that enhancement in TR might play a role in reorienting the metabolic fluctuations which occurred in response to BRs. Also, the changes in respiratory rate are directly associated with the changes in respiratory pathway capacity (Noguchi et al., 2001; Dinakar et al., 2010b). This flexibility in mETC makes plants adapt to various environmental conditions such as high light (Dinakar et al., 2010a), salt (Derevyanchuk et al., 2015), cold (Arfan et al., 2019) and drought (Hu et al., 2019). Thus, the addition of BL not only increased the TR in MCP but also significantly enhanced the capacity of the COX and AOX pathways (**Figures 2A**, **3A**). However, the AOX pathway capacity and the ratio of AOX pathway capacity to TR were significantly increased when compared with the COX pathway capacity and its ratio to TR (**Figure 2B**). This result is in corroboration with previous findings (Deng et al., 2015; Derevyanchuk et al., 2015; Zhu et al., 2016; Arfan et al., 2019; Derevyanchuk et al., 2019; Hu et al., 2019).

Similar to TR, an increase in rates of PCA and PSII is observed in MCP upon treatment with BL (**Figures 3B, C**). Further, this increase in PCA rates corroborated well with the rise in both maximal and actual activities and transcripts associated with CBC enzymes (NADP-GAPDH, FBPase, and PRK) examined in the present study (**Figures 3B**, **4**, **8A**). Many previous reports revealed that BRs stimulates the enzymatic activity/transcripts of redox-sensitive CBC enzyme through ROS such as H_2_O_2_ (Jiang et al., 2012b; Li et al., 2022). Further, the studies of Jiang et al. (2012b) demonstrated that BRs enhanced the activity of redox-sensitive CBC enzyme to promote CO_2_ assimilation by a modest rise in ROS levels and maintenance of cellular redox related to glutathione. Similarly to this study, BL caused a marginal rise in ROS levels, increased activity and transcripts of CBC enzymes, and reduced redox ratios of pyridine nucleotides (**Figures 4**, **6**, **7**, **8A**).

Besides, several studies showed an increase in PSII activity in response to BRs under both control (Lima and Lobato, 2017) and stress conditions such as salt (Zhu et al., 2016), cold (Arfan et al., 2019) and drought (Hu et al., 2019). This increase in PSII activity in the presence of BRs is attributed to the rise in the efficiency of light utilization and the dissipation of excitation energy at PS II antennae (Hu et al., 2013). The studies of Li et al. (2015) suggested that treatment with BRs increased the proportion of open PSII centers and thereby, the efficiency in capturing the light energy for the photochemical reactions.

### BL sustained the interdependence of photosynthesis and respiration in MCP

Any restriction in the transport of electrons through COX or AOX pathways caused over-reduction of mETC, leading to a decrease in various components associated with the process of respiration as well as photosynthesis (Padmasree and Raghavendra, 1999a; Padmasree and Raghavendra, 1999b; Padmasree and Raghavendra, 1999c; Padmasree and Raghavendra, 2001a; Dinakar et al., 2010b; Vishwakarma et al., 2015; Analin et al., 2020; Challabhathula et al., 2022). Similar to earlier reports, the restriction of COX and AOX pathways of mETC in MCP resulted in a decrease in TR, PSII activity, PCA, and activity/transcripts of CBC enzymes (**Figures 3**, **4**, **8A**).

Further, treatment of MCP with BL in the presence of AA or SHAM recovered the TR and PSII activity marginally but not to the extent observed in the absence of inhibitors (**Figures 3A, C**). This could be possible because only one pathway is inhibited while the other pathway of mETC is operative. Thus, under restricted electron transport through mitochondria, BL might try to maintain the flux of electrons either through COX or AOX to support the electron flow at the photochemical reaction centers of chloroplasts. The previous studies suggested that BRs might play a role in balancing the electron transfer from chloroplast to mitochondria through the AOX pathway to reduce the over-accumulation of ROS (Deng et al., 2015; Wei et al., 2015; Hu et al., 2019). Also, the studies of Jiang et al. (2013b) suggested that the positive effect of EBR on the recovery of PSII may be related to its impact on the activity of physiological metabolism, which provides ATP for the repair of D1 protein.

Further, adding BL to MCP in the presence of AA or SHAM also showed a significant decrease in the responses of PCA and activities/transcripts associated with redox-regulated CBC enzymes (**Figures 3B**, **4**, **8A**). However, this decline in PCA was more pronounced when BL treatment was superimposed with SHAM than AA. This result suggests that an over-reduced environment caused by inhibition in either COX or AOX pathways might severely affect the redox-regulated CBC enzymes at the biochemical and molecular levels, resulting in decreased expression and activity of CBC enzymes. Furthermore, adding BL in such an over-reduced condition might not relieve the injury to redox-regulated CBC enzymes, which play an essential role in PCA.

### BL keeps up malate valve and maintains a low cellular redox state in coordination with mETC to promote photosynthesis in MCP

During active photosynthesis, the photochemical reactions produce reducing equivalents at substantially exponential rates than the demand of CBC. Under this circumstance, the malate valve permits the exchange of malate for OAA across the chloroplast membrane to export extra reducing equivalents from chloroplasts to mitochondria *via* the cytosol through dicarboxylate transporters present in both chloroplasts and mitochondrial membranes (Scheibe, 2004; Selinski and Scheibe, 2019). An NADP-specific malate dehydrogenase (Chl-MDH) present in chloroplasts significantly transfers excess reducing equivalents from NADPH to OAA and generates malate to be transported to cytosol or mitochondria. Besides, in mitochondria, malate formed during the TCA cycle and imported through the malate valve is transformed into OAA by NAD-specific malate dehydrogenase (Dinakar et al., 2010a). The studies of Dinakar et al. (2016) observed a pronounced increase in malate levels under hyper-osmotic stress conditions in the presence of SHAM, depicting the role of malate in chloroplast and mitochondrial interactions. Besides, numerous reports showed that BRs maintain cellular redox homeostasis *via* increment in the ratios of reduced glutathione (GSH) to oxidized glutathione (GSSG) (Jiang et al., 2012b; Ramakrishna and Rao, 2013; Xia et al., 2018; Li et al., 2022).

In the present study, the addition of BL to MCP showed a decrease in the redox ratios related to both NADPH and NADH along with an increase in malate and transcripts associated with malate valve, i.e., *Chl-MDH* and *M-MDH* (**Figures 5B**, **6**, **8B**). These results suggest that BL participates in the crosstalk between chloroplast and mitochondria by modulating the malate valve to maintain cellular redox homeostasis and enhance photosynthesis. Also, the studies of Hu et al. (2019) showed an increment in the activity of both NADP-MDH and NAD-MDH upon treatment of leaves with EBL under drought. Further, an aggravation in a decrease of PCA along with an enhancement in the redox ratio of NADPH and NADH during treatment of MCP with BL in the presence of AA or SHAM confirm the significance of BRs along with mETC in keeping up the activity of malate valve and maintaining the cellular redox state at low levels to optimize chloroplastic photosynthesis (**Figures 3B**, **5B**, **6**, **8B**). Also, the present study reveals that BL could not overcome the negative effect of excess reducing equivalents on PCA, which is generated during disrupted mETC. However, the component of malate valve was active and maintained in response to BL. Thus, it might be trying to reverse or cope with the tremendous loss that occurred by metabolic fluctuation under restricted mETC.

### BL prevented excess ROS generation by activating AOX through malate valve and enhancing the capacity of mETC and antioxidant system

AOX regulates the cellular redox and ROS generation to optimize photosynthesis by modulating the malate valve (Padmasree and Raghavendra, 1999c; Yoshida et al., 2007; Dinakar et al., 2010a; Vishwakarma et al., 2015; Dinakar et al., 2016; Cheng et al., 2020; Garmash et al., 2020; Manbir et al., 2022; Challabathula et al., 2022). Further, malate formed in mitochondria gets utilized by the malic enzyme to form pyruvate (an activator of AOX). The studies of Day et al. (1995) demonstrated using isolated mitochondria that intra-mitochondrial pyruvate production and subsequent AOX activation are associated with malic enzyme activity. The studies of Sankar et al. (2022) demonstrated through molecular docking that Cys177 residue present in the binding pocket for pyruvate plays a role in regulating the activity of AtAOX1a post-translationally. Further, the studies of Dinakar et al. (2010a, 2016) illustrated that the pyruvate accumulated in response to stress (high light, osmotic, and temperature) enhanced the AOX pathway capacity. Also, adding BL to MCP resulted in an enhancement in pyruvate and malate, which might contribute to the enhancement of the AOX pathway capacity observed in the present study (**Figures 2**, **5B, C**). Thus, malate might be converted to pyruvate by NAD-ME, which activates the AOX pathway for oxidation of excess reducing equivalents and maintenance of ROS at an optimal level. Further, an aggravation in cellular ROS and redox ratios of NAD(P)H when BL was added to MCP in light under restricted electron transport through the AOX pathway confirms that BL prevents excess ROS generation by activating the AOX pathway through the malate valve during active photosynthesis (**Figures 2A**, **5B, C**, **6**, **7**, **8B**).

BR-induced ROS is also known to enhance the expression of the antioxidant system (Xia et al., 2009; Lima and Lobato, 2017). In the present study, BL increased the ROS marginally along with transcripts of the antioxidant system (**Figures 7**, **8C**). The studies of Fu et al. (2019) demonstrated that BRs improved cold tolerance in *Elymus nutans* by enhancing the activities of CAT, APX, GR, DHAR, and MDAR but not SOD. Also, under restricted COX or AOX pathway, over-accumulation of ROS was shown in *P. sativum* and *Arabidopsis*, which activated the antioxidant system (Dinakar et al., 2010b; Vishwakarma et al., 2015). The present study has shown that adding BL to MCP under disrupted mETC aggravated the cellular ROS and transcripts associated with the antioxidant system in parallel to the reduction in PCA and CBC enzyme activity/transcripts (**Figures 3B**, **4**, **7**, **8A, C**). Further, the minimal increase in the redox ratio of NADPH as compared to NADH under disrupted conditions of mETC suggests that NADPH might have been utilized in the ascorbate-glutathione cycle to minimize the H_2_O_2_ levels (**Figures 6**, **7**, **8C**). These results suggest that BL might play a role in preventing excess ROS generation by stimulating antioxidant activity along with mETC to optimize photosynthesis.

## Conclusion

The present study demonstrates the interaction between chloroplasts and mitochondria in response to BL for balancing carbon and redox energy. This BL-triggered interaction facilitates the reconfiguration of biochemical factors (ROS, pyruvate, malate, and redox ratios of pyridine nucleotides [NAD(P)H], resulting in the enhancement of TR, PCA, and PSII activity with a concomitant rise in activity/transcripts of redox-regulated CBC enzymes and the capacity of COX and AOX pathways. The results highlight the studies using COX or AOX pathway inhibitors to confirm the interactive role of BL with mETC in a coordinated manner for optimizing photosynthesis that is evident by a decrease in the PCA and CBC enzymes activity/transcripts, which in turn is caused due to imbalance in the ratios of reduced to oxidized pyridine nucleotides [NAD(P)H] leading to accumulation of ROS. Thus, BL prevents (i) over-reduction of the cellular environment by activating the AOX pathway through malate valve and (ii) excessive ROS generation by enhancing the capacity of COX and AOX pathways along with antioxidant systems in a photosynthesizing cell.

## Data availability statement

The original contributions presented in the study are included in the article. Further inquiries can be directed to the corresponding author.

## Author contributions

KP conceived the project. KM performed the experiments. KP and KM interpreted the data. KM wrote the manuscript. KP edited the manuscript. Both authors contributed to the article and approved the submitted version.

## Supplementary material

Supplementary Table 1 List of Primer used in Real Time-PCR studies:

## Glossary

**Table:**

AOX	Alternative oxidase
AA	Antimycin A
APX	Ascorbate peroxidase
BL	Brassinolide
BRs	Brassinosteroids
CAT	Catalase
CBC	Calvin-Benson cycle
CCCP	Carbonyl cyanide-3-chlorophenylhydrazone
Chl	Chlorophyll
COX	Cytochrome oxidase
DHAR	Dehydroascorbate reductase
EBR	Epibrassinolide
FBPase	Fructose-1,6-bisphosphatase
Glc-6-P	Glucose-6-Phosphate
GSH	Reduced glutathione
GSSG	Oxidized glutathione
GR	Glutathione reductase
H_2_DCFDA	2,7-dichlorofluorescein diacetate
KCN	Potassium cyanide
MDHAR	Monodehydroascorbate reductase
mETC	Mitochondrial electron transport chain
MCP	Mesophyll cell protoplasts
NAD	Oxidized nicotinamide adenine dinucleotide
NADH	Reduced nicotinamide adenine dinucleotide
NADP	Oxidized nicotinamide adenine dinucleotide phosphate
NADPH	Reduced nicotinamide adenine dinucleotide phosphate
NADP-GAPDH	NADP-dependent Glyceraldehyde-3-phosphate dehydrogenase
NADP-MDH (Chl-MDH)	(Chloroplastic-MDH), NADP-dependent Malate dehydrogenases
NAD-MDH (M-MDH)	(Mitochondrial-MDH), NAD-dependent Malate dehydrogenases
NAD-ME	NAD-dependent Malic Enzyme
OAA	Oxaloacetic acid
PCA	Photosynthetic carbon assimilation
PRK	Phosphoribulokinase
tAPX	Thylakoid ascorbate peroxidase
TCA	Tricarboxylic acid cycle
TR	Total respiration
RR	Residual respiration
ROS	Reactive oxygen species
sAPX	Stromal ascorbate peroxidase
SOD	Superoxide dismutase
SHAM	Salicylhydroxamic acid
