# Supplementary material for: Brassinolide promotes interaction between chloroplasts and mitochondria during the optimization of photosynthesis by the mitochondrial electron transport chain in mesophyll cell protoplasts of Arabidopsis thaliana
Source: Front Plant Sci. 2023 Apr 11;14:1099474. doi: 10.3389/fpls.2023.1099474 (PMC10126290; doi:10.3389/fpls.2023.1099474)
Supplement: Supplementary Table 1 — List of Primer used in Real Time-PCR studies: [file Table_1.docx]

**SUPPLEMENTARY TABLE**

**Table S1:**

Detailed list of sequence for forward and reverse primer of all the genes examined in the Real Time-PCR studies:

| **Gene name** | **Accession no.** | **Primer sequence (5' > 3')** | **Amplicon length (bp)** |
| --- | --- | --- | --- |
| *UBQ5* | AT3G62250 | F CCAAGCCGAAGAAGATCAAG  R ATGACTCGCCATGAAAGTCC | 149 |
| *GAPDH* | AT3G26650 | F TTTGCCTCCAAGTCATCAATTCTT  R AGCAGACAGGAGAGCAAAAAGGA | 78 |
| *FBPase* | AT1G43670 | F CTCGCCCAATTGCCCATCAC  R AGGCTGTTCACGGTCAGAGT | 80 |
| *PRK* | AT1G32060 | F TCACTCAACCTGAGCTCACAAAGC  R TTTGGTCTCCTCTTCTTCTCCGC | 70 |
| *Chl-MDH* | AT5G58330 | F ACCTCACTGGAGAGGGCATTG  R AATGTGGCTGTGCTCTGTACAGC | 135 |
| *M-MDH* | AT1G53240 | F TTGCCTCGAAGGTGAGGTTGG  R GAGGTGGCAACTCTAGCTGGA | 204 |
| *CSD1* | AT1G08830 | F TGAACTCAGCCTGGCTACTGG  R AGCCACACACCAGAAGATACACAC | 164 |
| *CAT1* | AT1G20630 | F TGGGATTCAGACAGGCAAGAACG  R GTTTGGCCTCACGTTAAGACGAGT | 162 |
| *sAPX* | AT4G08390 | F CCTCCGGAGGGTATCGTTATCTA  R ACAGCCAGAAACATTGTCCAAAAGG | 162 |
| *tAPX* | AT1G77490 | F TGGAGAAGCAGGAGGACAGT  R GCAGCCACATCTTCAGCATA | 177 |
| *MDHAR* | AT1G63940 | F CAAACTACTCTCATTGCCCTTTAGC  R GAAACAAGTGTGTGAGTCTCTCTGT | 70 |
| *DHAR* | AT1G19570 | F CCCACTGGTGGGTGGAGAAT  R CCTTTGTTTGCAGAGACGGATGA | 94 |
| *GR* | AT3G54660 | F TACCACTCTCGCCGGTTTTC  R TTCCGGCACCGATAGTGAAG | 97 |
